# Effect of Inhaled Nebulized Furosemide (40 and 120 mg) on Breathlessness during Exercise in the Presence of External Thoracic Restriction in Healthy Men

**DOI:** 10.3389/fphys.2018.00086

**Published:** 2018-02-12

**Authors:** Marcus Waskiw-Ford, Anne Wu, Amar Mainra, Noah Marchand, Abdullatif Alhuzaim, Jean Bourbeau, Benjamin M. Smith, Dennis Jensen

**Affiliations:** ^1^Clinical Exercise and Respiratory Physiology Laboratory, Department of Kinesiology and Physical Education, McGill University, Montréal, QC, Canada; ^2^Department of Medicine, Respiratory Division, McGill University, Montréal, QC, Canada; ^3^Respiratory Epidemiology and Clinical Research Unit, Montréal Chest Institute, McGill University Health Centre, Montréal, QC, Canada; ^4^Meakins-Christie Laboratories, Research Institute of the McGill University Health Centre, Montréal, QC, Canada; ^5^Centre for Outcomes Research and Evaluation, Research Institute of the McGill University Health Center, Montréal, QC, Canada; ^6^Translational Research in Respiratory Diseases Program, Research Institute of the McGill University Health Center, Montréal, QC, Canada; ^7^Research Centre for Physical Activity and Health, McGill University, Montréal, QC, Canada

**Keywords:** furosemide, breathlessness, external thoracic restriction, exercise, pulmonary stretch receptor

## Abstract

Inhalation of nebulized furosemide has been shown to alleviate breathlessness provoked experimentally in health and disease; however, it remains unclear whether the efficacy of nebulized furosemide on breathlessness is dose-dependent. We tested the hypothesis that inhaled nebulized furosemide would be associated with a dose-dependent relief of breathlessness during exercise testing in the setting of abnormal restrictive constraints on tidal volume (V_T_) expansion. In a randomized, double-blind, crossover study, 24 healthy men aged 25.3 ± 1.2 years (mean ± SE) completed a symptom-limited constant-load cycle endurance exercise test in the setting of external thoracic restriction *via* chest wall strapping to reduce vital capacity by ~20% following single-dose inhalation nebulized furosemide (40 and 120 mg) and 0.9% saline. Compared with 0.9% saline, neither 40 nor 120 mg of inhaled nebulized furosemide had an effect on ratings of perceived breathlessness during exercise or an effect on cardiometabolic, ventilatory, breathing pattern, or dynamic operating lung volume responses during exercise. Urine production rate, the percentage of participants reporting an “urge to urinate” and the intensity of perceived “urge to urinate” were all significantly greater after inhaling the 120 mg furosemide solution compared with both 0.9% saline and 40 mg furosemide solutions. We concluded that, under the experimental conditions of this study, inhalation of nebulized furosemide at doses of 40 and 120 mg did not alleviate breathlessness during exercise in healthy men.

## Introduction

The population-based prevalence of adults reporting breathlessness that limits activities of daily life is ~10% (Currow et al., 2009; Bowden et al., 2011; Ekström et al., 2016). Breathlessness is ubiquitous in advanced disease across a range of both malignant and non-malignant diagnoses (Ekström et al., 2016); for example, Mullerova et al. (2014) reported that >75% of adults with advanced chronic obstructive pulmonary disease (COPD) experienced physical activity-limiting breathlessness. Notwithstanding the high prevalence and burden of breathlessness in the general population and among adults with advanced disease, effective management of this symptom remains a challenge for healthcare providers. For instance, Sundh and Ekström (2016) found that 57% of adults with COPD experienced persistent and disabling physical activity-related breathlessness despite treatment of their underlying pathophysiology with inhaled triple therapy. With the possible exception of low-dose systemic opioids (Ekström et al., 2015; Barnes et al., 2016), which are rarely prescribed for relief of breathlessness (Ahmadi et al., 2016), there is currently no evidence-based pharmacotherapy indicated for use in the management of chronic breathlessness syndrome, a distinct clinical entity recently defined as breathlessness that persists despite optimal treatment of the underlying pathophysiology and that results in disability (Johnson et al., 2017).

Several studies have demonstrated that inhalation of nebulized furosemide (40 mg) compared with nebulized 0.9% saline decreased intensity ratings of perceived breathlessness provoked by a variety of respiratory stimuli at rest in healthy adults (Nishino et al., 2000; Minowa et al., 2002; Moosavi et al., 2007) or by constant-load cycle endurance exercise testing in COPD (Ong et al., 2004; Jensen et al., 2008). A randomized, double-blind, parallel group study by Sheikh Motahar Vahedi et al. (2013) similarly reported that nebulized furosemide (40 mg) was superior to nebulized 0.9% saline as an adjunct to conventional therapies for alleviating breathlessness at rest in adults admitted to the emergency department with an acute exacerbation of COPD.

Although the mechanisms underlying relief of breathlessness with nebulized furosemide remain unclear, changes in the activity of pulmonary stretch receptors (PSRs) that provide sensory feedback information on lung expansion *via* the vagus nerve to cortical and subcortical regions of the brain implicated in the perception of breathlessness are likely contributory (Davenport and Vovk, 2009; Nishino, 2009). To this end, Sudo et al. (2000) showed that inhalation of nebulized furosemide enhanced the activity of slowly adapting PSRs (SARs) and suppressed the activity of rapidly adapting PSRs (RARs) during lung inflation in anesthetized rats. In keeping with these observations, Nehashi et al. (2001) reported that nebulized furosemide inhibited respiratory distress induced by airway occlusion in anesthetized cats. Sakurai et al. (1998) similarly reported that lung expansion inhibited respiratory distress induced by airway occlusion in a dose-related manner and that bilateral vagotomy totally abolished this effect, presumably *via* loss of sensory feedback from PSRs. On the basis of these observations and the purported role of PSRs in the neuromodulation of breathlessness in humans (Manning et al., 1992; Flume et al., 1996; Vovk and Binks, 2007), it has been proposed that nebulized furosemide alleviates breathlessness by altering pulmonary vagal afferent activity from PSRs, presumably mimicking greater tidal volume (V_T_) expansion (Nishino et al., 2000; Sudo et al., 2000; Nehashi et al., 2001; Moosavi et al., 2007; Nishino, 2009).

However, relief of breathlessness following inhalation of nebulized furosemide is not a universal finding, with a growing number of studies reporting no statistically significant effect of nebulized furosemide (40–80 mg) compared with nebulized 0.9% saline on intensity ratings of perceived breathlessness: during arm exercise tests in symptomatic adults with lung cancer or mesothelioma (Wilcock et al., 2008); at rest in sulfur mustard gas-exposed adults with irreversible obstructive airway disease (Panahi et al., 2008); during expiratory flow-limited incremental cycle exercise testing in healthy adults (Laveneziana et al., 2008); and during the combination of hypercapnia and constrained ventilation in healthy adults (Banzett et al., 2018; Morelot-Panzini et al., 2018). Thus, the efficacy of nebulized furosemide on breathlessness remains uncertain and requires further investigation. In particular, it remains unclear whether the efficacy of inhaled nebulized furosemide on breathlessness is dose-dependent.

The objective of this randomized, double-blind, crossover study was to examine the acute effect of nebulized furosemide at doses of 40 and 120 mg on exertional breathlessness in healthy men. Considering the possibility that nebulized furosemide alleviates breathlessness by mimicking greater V_T_ expansion *via* altered pulmonary vagal afferent activity from PSRs, we hypothesized that, compared with nebulized 0.9% saline, nebulized furosemide would be associated with a dose-dependent relief of breathlessness during constant-load cycle endurance exercise testing in the presence of abnormal restrictive constraints on V_T_ expansion.

## Methods

### Study design

This was a single-center, randomized, double-blind, crossover study (ClinicalTrials.gov identifier: NCT01851980) wherein participants visited the laboratory on four separate occasions over a period of 2–4 weeks. Visits were conducted at approximately the same time of day (±1-h) for each participant and separated by ≥48-h to minimize the possibility of a “carry-over” effect between *Visits 2, 3, and 4* (i.e., treatment periods A, B, and C). Participants were instructed to avoid strenuous exercise and alcohol on each test day. The study protocol and informed consent form received regulatory approval from Health Canada (File No. HC6-24-c193768) and ethics approval from the Research Ethics Board of the Research Institute of the McGill University Health Centre (15-370-MUHC). All participants gave written informed consent in accordance with the Declaration of Helsinki.

### Participants

Participants included healthy, non-smoking, non-obese (body mass index <30 kg/m^2^) men aged 18–40 years with a forced expiratory volume in 1-s (FEV_1_) ≥80% predicted (Quanjer et al., 2012) and a FEV_1_-to-forced vital capacity ratio (FEV_1_/FVC) ≥70%. Participants were excluded if they: had a known or suspected cardiovascular, metabolic, pulmonary, musculoskeletal, endocrine, and/or neuromuscular disorder; were taking physician prescribed medications; were allergic or hypersensitive to furosemide and/or any other sulfonamide-derived medication(s); had an anion gap of <10 or >16 mEq/L at rest (Klaestrup et al., 2011); were hypokalemic, defined as an arterialized capillary blood [K^+^] of <3.5 mEq/L at rest; and/or were severely hypotensive, defined as a systolic blood pressure of ≤90 mmHg and/or diastolic blood pressure of ≤60 mmHg at rest.

*Visit 1* included: screening for eligibility criteria; routine clinical assessment of heart rate and rhythm by 12-lead electrocardiography (GE Marquette's CardioSoft® 12-lead ECG system; CareFusion, Yorba Linda, CA), blood pressure by automated sphygmomanometer (Carescape™ V100 Dynamap® monitor; GE Healthcare, Freidburg, Germany) and oxyhemoglobin saturation by finger pulse oximeter (Carescape™ V100 Dynamap® monitor); collection of arterialized capillary blood samples from a warmed earlobe (Finalgon® Cream, Boehringer Ingelheim GmbH) into a pre-heparinized capillary tube (safeCLINITUBES, D957P-70-125; Radiometer Copenhangen, Denmark) for measurement of [K^+^], [Na^+^], [Cl^−^], and [${\text{HCO}}_{3}^{-}$]—and subsequent calculation of the anion gap [i.e., ([Na^+^] + [K^+^])–([Cl^−^] + [${\text{HCO}}_{3}^{-}$])]—using an OPTI^TM^ CCA-TS2 analyzer (OPTI Medical Systems Inc., Roswell, GA, USA); pulmonary function testing, including spirometry and slow vital capacity (SVC) maneuvers; external thoracic restriction by chest wall strapping (CWS) to reduce SVC by ~20% of its baseline (unrestricted) value at rest (Mendonca et al., 2014; Kotrach et al., 2015); spirometry and SVC maneuvers after ~5-min of acclimatization to the CWS; and a symptom-limited incremental cardiopulmonary cycle exercise test (CPET) in the presence of CWS to determine peak power output (PPO) as well as to familiarize participants to CPET with CWS.

After randomization of treatments (*Visits 2–4*) according to a computer-generated block randomization schedule prepared by an independent third-party, participants inhaled a 12 ml solution containing either 0.9% saline (12 ml), 40 mg of furosemide [4 ml of 10 mg/ml furosemide (Sandoz, Boucherville, QC, Canada) + 8 ml of 0.9% saline] or 120 mg of furosemide (12 ml of 10 mg/ml furosemide) administered by means of an Omron® NE-C30 CompAir® Elite Compact Compressor Nebulizer (Omron Healthcare, Inc., Blannockburn, IL, USA) that produced particles with a mass median diameter of ~5 μm at a nebulization rate of ~0.35 ml/min. During nebulization, participants were instructed to take deep and slow breaths through a pediatric (open) facemask that surrounded the mouth with nasal passages occluded by a nose clip. Within ≤10-min after nebulization, participants were fitted with the CWS, which was adjusted to decrease SVC to within ±10% of the value recorded prior to CPET at *Visit 1*. After ~5-min of acclimatization to the CWS, participants performed spirometry and SVC maneuvers, followed immediately thereafter by a symptom-limited constant-load CPET at 80% of the PPO determined at *Visit 1*. Following ~10-min of recovery from CPET, participants performed two SVC maneuvers to determine whether the CWS had loosened during CPET.

To help mask the taste of the nebulized solutions and promote blinding of treatments, participants rinsed their mouth with an alcohol-free mint flavored mouthwash for 20-s immediately before the start of nebulization and after ~20-min (i.e., midpoint) of nebulization. Participants were instructed to empty their bladder immediately before inhaling the nebulized solutions. Following inhalation of the nebulized solutions, participants were asked to empty their bladder into a urine collection container for determination of urine production rate [an index of diuresis and calculated as cumulative urine volume (ml) ÷ total duration of the observation period (min) beginning at the start of nebulization] immediately before the start of CPET, following 30-min of recovery from CPET and/or whenever necessary. Immediately after inhaling the nebulized solutions, participants were asked, “Do you have an urge to urinate?” If “yes,” participants rated the intensity of their perceived “urge to urinate” using a 100-mm visual analog scale (VAS), where “0” represented “absolutely no urge to urinate” and “100” represented “the most intense urge to urinate imaginable or ever experienced.” If “no,” the intensity of perceived “urge to urinate” was assumed to be “0.” Upon completing all study-related procedures at the end of *Visit 4*, participants were asked to identify during which visit they believed that they inhaled the 0.9% saline, 40 mg furosemide and 120 mg furosemide solutions.

### Pulmonary function tests

Spirometry and SVC maneuvers were performed with participants seated using automated equipment (Vmax Encore^TM^ 29c; CareFusion, Yorba Linda, CA) according to recommended techniques (Miller et al., 2005a,b).

### External thoracic restriction

An inelastic strap (Nike Structured Strength Training Belt; Beaverton, OR, USA) was fitted just beneath the axillae and around the chest to envelope the rib cage (Mendonca et al., 2014; Kotrach et al., 2015). The desired degree of lung volume restriction was achieved by tightening a Velcro strap at the back of the CWS while participants expired to residual volume, followed shortly thereafter by a series of SVC maneuvers.

### Cardiopulmonary exercise testing

Exercise tests were performed on an electronically braked cycle ergometer (Lode Corival; Lode B.V. Medical Tech., Groningen, The Netherlands) using a computerized CPET system (Vmax Encore^TM^ 29c). Incremental CPETs consisted of a steady-state rest period of ≥6-min, followed by 25 W increases in power output (starting at 25 W) every 2-min: PPO was defined as the highest power output that the participant was able to sustain for ≥30-s. Constant-load CPETs consisted of a steady-state rest period of ≥6-min, followed by a 1-min warm-up at 25% of PPO and then a step increase in power output to 80% of PPO. During both incremental and constant-load CPETs, pedal cadence was maintained between 60 and 90 rev/min and participants were verbally encouraged to exercise to the point of symptom-limitation.

Standard respiratory and gas exchange parameters were collected breath-by-breath while participants breathed through a rubber mouthpiece and low-resistance flow transducer with nasal passages occluded by a nose clip. Heart rate and rhythm were monitored by 12-lead electrocardiography (GE Marquette's CardioSoft® 12-lead ECG system), while oxyhemoglobin saturation was monitored by finger pulse oximeter (Carescape^TM^ V100 Dynamap® monitor). Inspiratory capacity (IC) maneuvers were performed at rest, within the last 30-s of every 2-min interval during CPET and at end-exercise. Assuming that total lung capacity does not change during CPET with CWS in healthy men, changes in IC and inspiratory reserve volume [IRV = IC–V_T_] reflect changes in dynamic end-expiratory and end-inspiratory lung volume, respectively.

Using Borg's modified 0–10 category ratio scale (CR10) (Borg, 1982), participants rated the intensity and unpleasantness of their perceived breathlessness as well as the intensity of their perceived leg discomfort and chest tightness at rest, within the last 30-s of every 2-min interval during CPET and at end-exercise. Prior to each CPET, participants were familiarized with Borg's CR10 scale and its endpoints were anchored such that “0” represented “no intensity (unpleasantness) at all” and “10” represented “the most severe intensity (unpleasantness) you have ever experienced or could ever imagine experiencing.” In addition, a script derived from Price et al. (1983) was read to each participant prior to each CPET to help distinguish between the intensity and unpleasantness of breathlessness. Participants verbalized their main reason(s) for stopping exercise; quantified the percentage contribution of breathlessness, leg discomfort and chest tightness to exercise cessation; and identified qualitative phrases that best described their breathlessness at end-exercise (O'Donnell et al., 2000).

### Analysis of exercise end-points

All physiological parameters were averaged in 30-s intervals at rest and during CPET. These parameters, averaged over the first 30-s of the 2nd min of every 2-min interval during CPET, were linked with IC and symptom measurements collected during the last 30-s of the same minute. Three main time points were used for the evaluation of measured parameters: (1) *pre-exercise rest*, defined as the average of the last 60-s of the steady-state period after ≥3-min of breathing on the mouthpiece while seated on the cycle ergometer before the start of CPET; (2) *isotime*, defined as the average of the first 30-s of the 2nd min of the highest equivalent 2-min interval of constant-load CPET completed by a given participant; and (3) *peak exercise*, defined as the average of the last 30-s of loaded pedaling during constant-load CPET at 80% of PPO. Exercise endurance time (EET) was defined as the duration of loaded pedaling during constant-load CPET at 80% of PPO.

### Sample size estimation and statistical analyses

Using a formula for a balanced analysis of variance (ANOVA) with crossover design in combination with Tukey's HSD adjustment method (Lenth, 2009), we estimated that 24 participants were needed to detect a ±1 Borg CR10 unit difference in breathlessness intensity ratings during constant-load CPET at isotime across the three treatments, assuming a two-tailed test of significance, a within-subject standard deviation of ±1 Borg CR10 units, α = 0.05 and β = 0.80.

A one-way repeated measures ANOVA with Tukey's HSD post-hoc test was used to examine the effect of treatment with 0.9% saline, 40 mg furosemide and 120 mg furosemide on: the duration of nebulization; the amount of time between the end of nebulization and the end of CPET; post-dose SVC and spirometric pulmonary function test parameters recorded prior to CPET; urine production rate; intensity ratings of the perceived “urge to urinate”; the percentage contribution of breathlessness, leg discomfort and chest tightness to exercise cessation; and EET. A two-way repeated measures ANOVA with Tukey's HSD post-hoc test was used to examine treatment, time and treatment^*^time effects on physiological and perceptual parameters measured at rest and during constant-load CPETs. Fisher's exact test was used to examine the effect of treatment with (i) 0.9% saline vs. 40 mg furosemide, (ii) 0.9% saline vs. 120 mg furosemide, and (iii) 40 mg furosemide vs. 120 mg furosemide on the percentage of participants reporting an “urge to urinate” after inhaling the nebulized solution. A chi-squared test was used to compare the selection frequency of the individual reasons for stopping exercise as well as the selection frequency of the individual descriptors of breathlessness at end-exercise across treatments. Analyses were performed using SigmaStat® (Version 3.5; Systat® Software, San Jose, CA, USA) and statistical significance was set at *p* < 0.05. Data are presented as means ± SE.

## Results

### Participant characteristics

Participants included 24 healthy, non-obese (body mass index, 23.9 ± 0.6 kg/m^2^) men aged 25.3 ± 1.2 years with normal spirometry (FEV_1_, 99 ± 3% predicted, z-score −0.13 ± 0.22; FEV_1_/FVC, 79.7 ± 1.3%, z-score −0.78 ± 0.19) and a symptom-limited PPO and peak rate of oxygen uptake (VO_2peak_) on incremental CPET with CWS of 205 ± 10 W and 41.9 ± 1.9 ml/kg/min, respectively.

### Time course of nebulization and post-dose CPET

The duration of nebulization was not significantly different across treatments: 0.9% saline, 35.7 ± 1.0 min; 40 mg furosemide, 36.4 ± 1.2 min; and 120 mg furosemide, 39.2 ± 1.1 min (*p* = 0.075). The amount of time between the end of nebulization and the end of CPET was not significantly different across treatments: 0.9% saline, 50.8 ± 1.3 min; 40 mg furosemide, 53.2 ± 1.4 min; and 120 mg furosemide, 55.3 ± 1.7 min (*p* = 0.086).

### Diuresis

**Figure 1:**
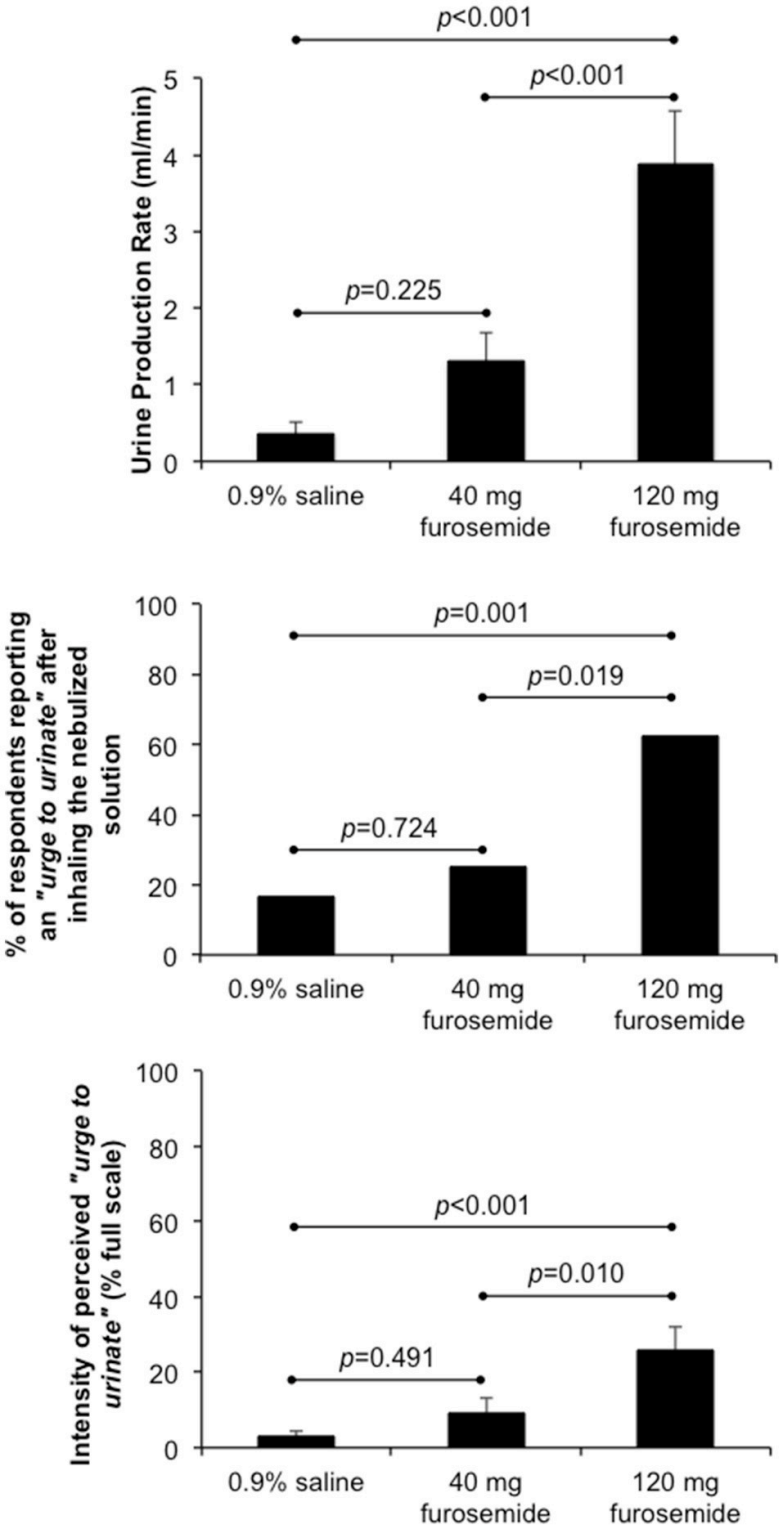
Effect of single-dose inhalation of nebulized furosemide (40 and 120 mg) on urine production rate (mean ± SE), the percentage of participants reporting an “urge to urinate” and the intensity of perceived “urge to urinate” (mean ± SE). Cumulative urine output was 37.8 ± 15.2, 135.8 ± 39.9, and 423.9 ± 77.3 ml over an observation period of 98.5 ± 1.4 min, 101.0 ± 1.8 min, and 107.6 ± 2.5 min during 0.9% saline, 40 mg furosemide and 120 mg furosemide treatment visits, respectively.

As illustrated in Figure 1, inhalation of the 40 mg furosemide solution had no statistically significant effect on urine production rate, the percentage of participants reporting an “urge to urinate” and/or the intensity of perceived “urge to urinate” compared with 0.9% saline. By contrast, urine production rate, the percentage of participants reporting an “urge to urinate” and the intensity of perceived “urge to urinate” were all significantly greater after inhaling the 120 mg furosemide solution compared with both 0.9% saline and 40 mg furosemide solutions. No other systemic or adverse effects were reported following inhalation of the 40 and 120 mg furosemide solutions.

### Effect of external thoracic restriction and nebulized furosemide on pulmonary function test parameters

The effect of CWS and nebulized furosemide on SVC and spirometric pulmonary function test parameters are presented in Table 1. By design, CWS decreased SVC recorded prior to CPET by 21 ± 1% (range: −15 to −31%), 22 ± 1% (range: −13 to −31%), 21 ± 1% (range: −15 to −31%), and 21 ± 1% (range: −13 to −33%) of its baseline (unrestricted) value at *Visit 1* and at the 0.9% saline, 40 mg furosemide, and 120 mg furosemide treatment visits, respectively. The SVC values recorded prior to CPET at the 0.9% saline, 40 mg furosemide, and 120 mg furosemide visits were closely matched to the target SVC value recorded prior to CPET at *Visit 1*: 99 ± 1% (range: 94–105%; *p* = 0.092 by paired *t*-test); 100 ± 1% (range: 95–111%; *p* = 0.810 by paired *t*-test); and 100 ± 1% (range: 95–109%; *p* = 0.769 by paired *t*-test), respectively. The intra-subject, between-day (or between-treatment) coefficient of variability in the SVC value recorded prior to CPET was 2.2 ± 0.2% (range: 0.7–4.5%). The SVC values recorded before vs. after CPET were not significantly different at *Visit 1* (4.41 ± 0.16 L vs. 4.54 ± 0.19 L; *p* = 0.386 by paired *t*-test) and at the 0.9% saline (4.36 ± 0.15 L vs. 4.47 ± 0.16 L; *p* = 0.106 by paired *t*-test), 40 mg furosemide (4.41 ± 0.16 L vs. 4.47 ± 0.16 L; *p* = 0.115 by paired *t*-test) and 120 mg furosemide visits (4.40 ± 0.15 L vs. 4.42 ± 0.15 L; *p* = 0.106 by paired *t*-test). Compared with 0.9% saline, neither dose of nebulized furosemide had an effect on SVC and spirometric pulmonary function test parameters recorded prior to CPET (Table 1).

**Table 1:** Effect of external thoracic restriction and inhaled nebulized furosemide on slow vital capacity (SVC) and spirometric pulmonary function test parameters.

**Parameter**	**Unrestricted**	**Visit 1**	**0.9% saline**	**40 mg furosemide**	**120 mg furosemide**
SVC, L	5.59 ± 0.19	4.41 ± 0.16	4.36 ± 0.15	4.41 ± 0.16	4.40 ± 0.15
FVC, L	5.48 ± 0.18	4.28 ± 0.14	4.25 ± 0.15	4.28 ± 0.14	4.26 ± 0.14
FEV_1_, L	4.34 ± 0.13	3.39 ± 0.09	3.46 ± 0.12	3.49 ± 0.11	3.48 ± 0.11
FEV_1_/FVC, %	79.7 ± 1.3	79.9 ± 1.5	81.8 ± 1.5	82.2 ± 1.4	82.1 ± 1.5
PEF, L/s	8.06 ± 0.28	6.54 ± 0.22	7.21 ± 0.22	7.43 ± 0.23	7.10 ± 0.24
FEF_25−75%_, L/s	4.25 ± 0.19	3.38 ± 0.15	3.46 ± 0.19	3.57 ± 0.18	3.60 ± 0.20

*Values are means ± SE. FVC, forced vital capacity; FEV_1_, forced expiratory volume in 1 s; PEF, peak expiratory flow; FEF_25−75%_, forced expiratory flow between 25 and 75% of the forced vital capacity maneuver*.

### Effect of nebulized furosemide on perceptual and physiological responses to CPET

**Figure 2:**
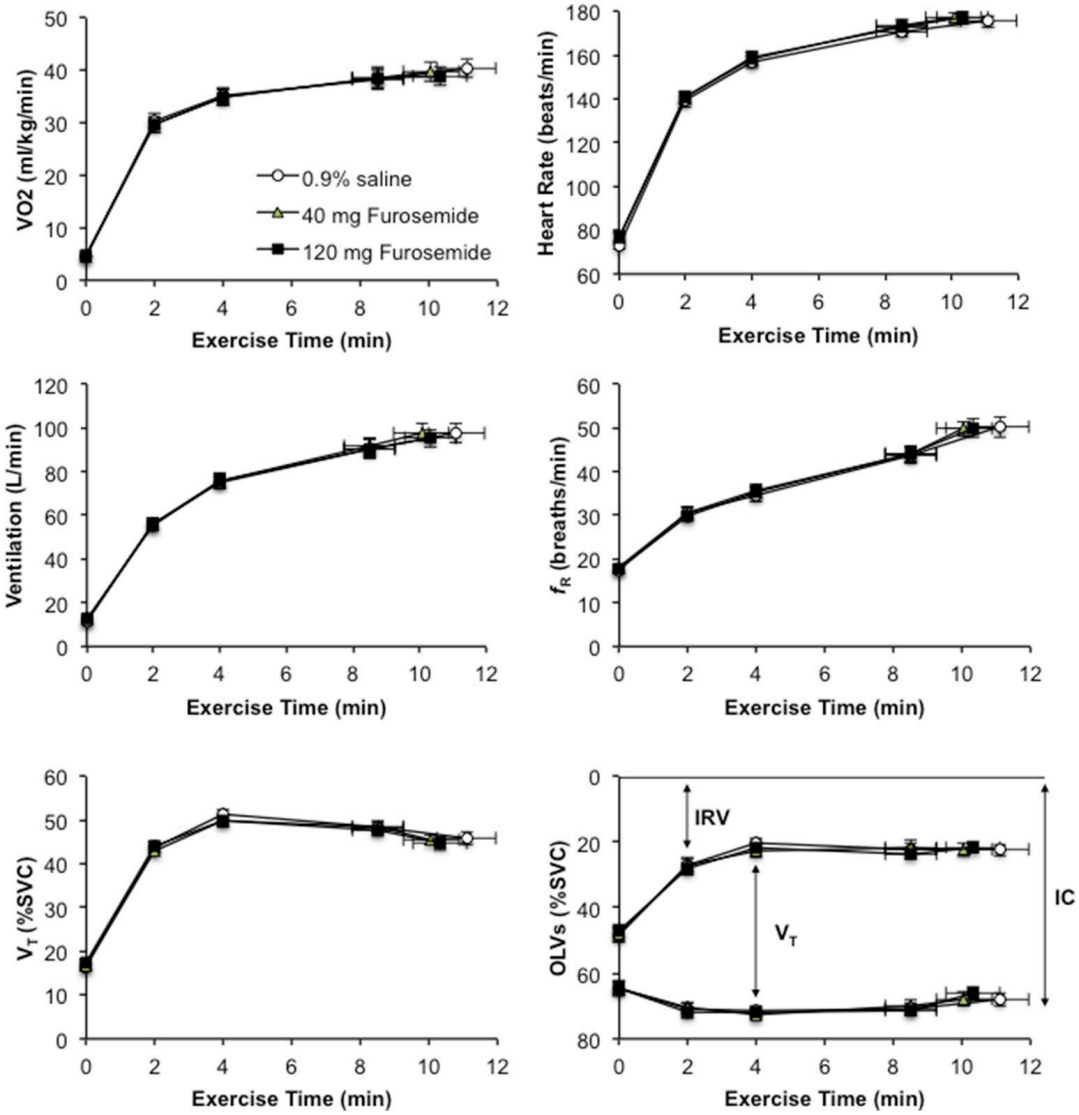
Effect of inhaled nebulized furosemide at doses of 40 and 120 mg on metabolic, cardiac, ventilatory, breathing pattern, and dynamic operating lung volume parameters at rest and during constant-load cycle endurance exercise testing at 80% of the symptom-limited peak power output achieved during incremental cycle exercise testing in the presence of external thoracic restriction, equivalent to 166 ± 8 W. VO_2_, rate of oxygen uptake; *f*_R_, respiratory frequency; V_T_, tidal volume; SVC, slow vital capacity measured prior to the start of exercise; OLVs, operating lung volumes; IRV, inspiratory reserve volume; IC, inspiratory capacity. Values are means ± SE at rest, at standardized submaximal time points of 2, 4, and 8.5 ± 0.8-min (isotime), and at peak exercise.

**Figure 3:**
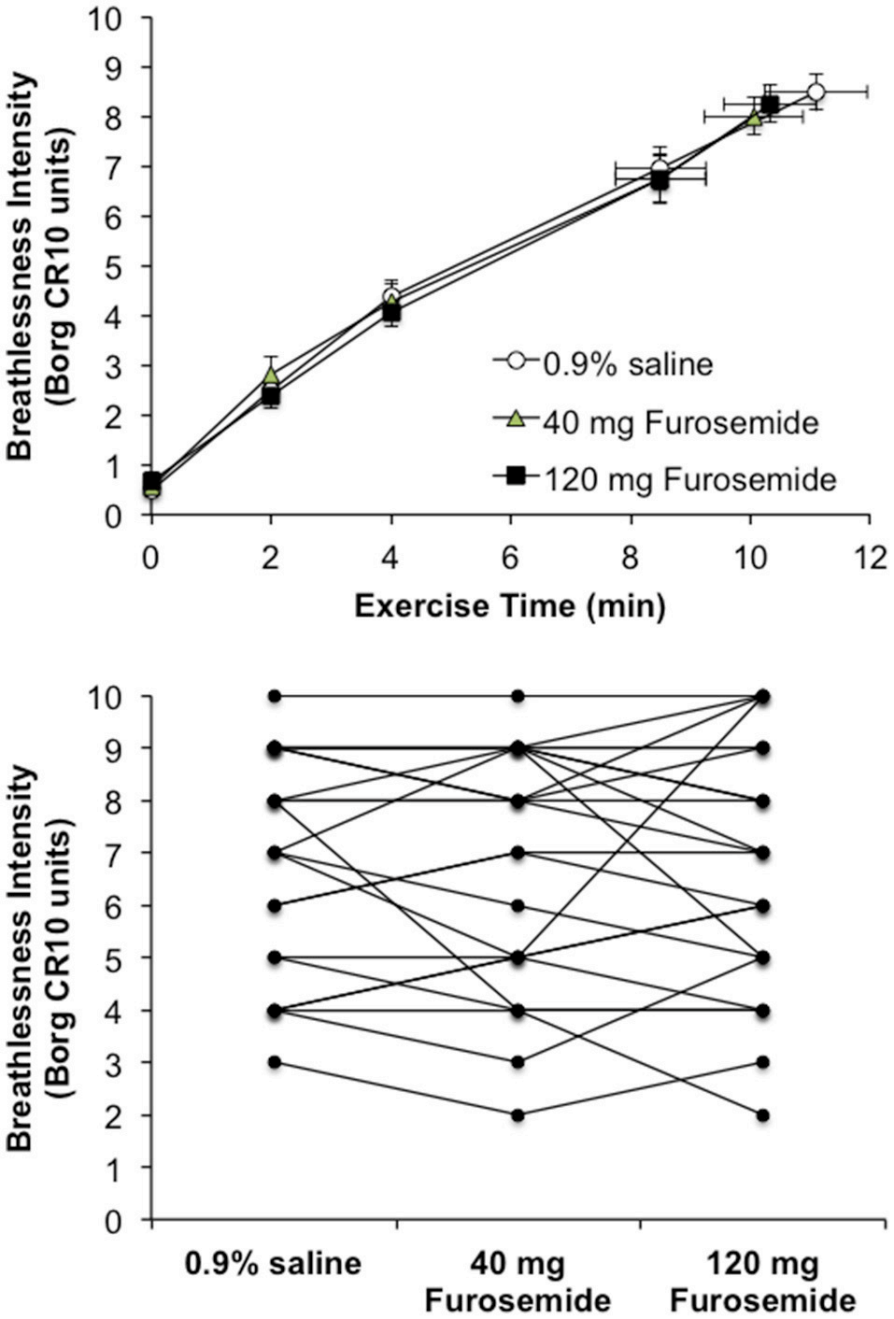
Effect of inhaled nebulized furosemide at doses of 40 and 120 mg on intensity ratings of perceived breathlessness at rest and during constant-load cycle endurance exercise testing at 80% of the symptom-limited peak power output achieved during incremental cycle exercise testing in the presence of external thoracic restriction, equivalent to 166 ± 8 W. **(Top)**: Values are means ± SE at rest, at standardized submaximal time points of 2, 4, and 8.5 ± 0.8-min (isotime), and at peak exercise. **(Bottom)**: Individual participant post-dose breathlessness intensity ratings during exercise at isotime.

Compared with 0.9% saline, neither dose of nebulized furosemide had an effect on EET or an effect on perceptual and physiological parameters recorded at rest and during CPET (Table 2, Figures 2, 3).

**Table 2:** Effect of inhaled nebulized furosemide on physiological and perceptual parameters at rest and during constant-load cycle endurance exercise testing at 80% of the symptom-limited peak power output achieved during incremental cycle exercise testing in the presence of external thoracic restriction, equivalent to 166 ± 8 W.

**Parameter**	**REST**			**ISOTIME**			**PEAK**		
	**0.9% saline**	**40 mg furosemide**	**120 mg furosemide**	**0.9% saline**	**40 mg furosemide**	**120 mg furosemide**	**0.9% saline**	**40 mg furosemide**	**120 mg furosemide**
Exercise time, min	–	–	–	8.5 ± 0.8	8.5 ± 0.8	8.5 ± 0.8	11.1 ± 0.8	10.1 ± 0.9	10.3 ± 0.8
Breathlessness intensity, Borg CR10 units	0.5 ± 0.2	0.6 ± 0.2	0.7 ± 0.2	7.0 ± 0.4	6.8 ± 0.5	6.8 ± 0.5	8.5 ± 0.4	8.0 ± 0.4	8.3 ± 0.4
Breathlessness unpleasantness, Borg CR10 units	0.5 ± 0.2	0.8 ± 0.2	0.7 ± 0.2	6.7 ± 0.5	6.8 ± 0.5	6.6 ± 0.5	8.2 ± 0.4	7.8 ± 0.5	7.9 ± 0.4
Leg discomfort, Borg CR10 units	0.4 ± 0.2	0.4 ± 0.1	0.3 ± 0.1	8.2 ± 0.4	8.0 ± 0.3	7.7 ± 0.4	9.5 ± 0.2	9.0 ± 0.3	8.8 ± 0.4
Chest tightness, Borg CR10 units	1.4 ± 0.2	1.8 ± 0.2	1.7 ± 0.3	6.6 ± 0.5	6.3 ± 0.5	6.6 ± 0.5	7.6 ± 0.4	7.3 ± 0.4	7.8 ± 0.4
VO_2_, ml/kg/min	4.4 ± 0.2	4.5 ± 0.2	4.7 ± 0.2	38.4 ± 1.8	38.6 ± 1.9	38.5 ± 2.0	40.5 ± 1.8	39.7 ± 1.9	39.0 ± 1.8
HR, beats/min	73 ± 2	76 ± 3	77 ± 2	171 ± 3	174 ± 2	173 ± 3	176 ± 3	177 ± 3	177 ± 3
O_2_ pulse, ml O_2_/beat	4.5 ± 0.2	4.5 ± 0.3	4.6 ± 0.3	17.7 ± 1.2	18.2 ± 1.3	18.2 ± 1.2	17.9 ± 1.2	18.0 ± 1.3	18.2 ± 1.2
V_E_/VCO_2_	43.4 ± 1.4	43.6 ± 1.3	42.3 ± 1.4	30.1 ± 0.6	30.2 ± 0.5	30.3 ± 0.6	32.2 ± 0.7	32.1 ± 0.5	32.1 ± 0.5
P_ET_CO_2_, mmHg	35.1 ± 0.6	35.2 ± 0.5	34.7 ± 0.6	36.0 ± 0.6	35.9 ± 0.5	35.8 ± 0.6	33.8 ± 0.6	33.8 ± 0.5	34.0 ± 0.5
SpO_2_, %	98.3 ± 0.3	98.0 ± 0.3	98.0 ± 0.3	97.2 ± 0.4	97.3 ± 0.4	97.6 ± 0.4	97.0 ± 0.4	97.0 ± 0.3	97.0 ± 0.4
V_E_, L/min	11.7 ± 0.5	12.2 ± 0.4	12.6 ± 0.8	90.2 ± 4.2	91.6 ± 4.2	90.6 ± 4.0	97.3 ± 4.4	97.9 ± 4.1	95.1 ± 3.9
V_T_, L	0.71 ± 0.03	0.72 ± 0.04	0.77 ± 0.06	2.12 ± 0.1	2.14 ± 0.1	2.10 ± 0.1	2.00 ± 0.10	2.00 ± 0.10	1.97 ± 0.10
V_T_, %SVC	16.4 ± 0.7	16.5 ± 0.8	17.3 ± 0.9	48.6 ± 1.2	48.4 ± 1.1	47.68 ± 1.3	45.81 ± 1.4	45.48 ± 1.1	44.62 ± 1.5
*f*_R_, breaths/min	17.5 ± 0.8	18.2 ± 0.9	17.6 ± 0.9	43.6 ± 1.9	43.9 ± 1.8	43.7 ± 1.6	50.1 ± 2.3	49.8 ± 1.6	50.0 ± 2.1
IC, L	2.82 ± 0.13	2.85 ± 0.14	2.85 ± 0.15	3.08 ± 0.12	3.01 ± 0.11	3.14 ± 0.12	2.97 ± 0.13	2.91 ± 0.10	2.91 ± 0.13
IC, %SVC	64.8 ± 2.1	64.7 ± 2.0	64.4 ± 2.2	70.7 ± 1.8	70.2 ± 2.2	71.3 ± 1.6	68.0 ± 2.0	67.9 ± 2.1	66.2 ± 2.1
Change in IC from rest, L	–	–	–	0.26 ± 0.09	0.26 ± 0.06	0.29 ± 0.08	0.16 ± 0.08	0.16 ± 0.06	0.06 ± 0.11
IRV, L	2.11 ± 0.12	2.12 ± 0.13	2.07 ± 0.12	0.96 ± 0.08	0.89 ± 0.09	1.03 ± 0.07	0.97 ± 0.09	0.93 ± 0.08	0.93 ± 0.08
IRV, %SVC	48.4 ± 2.1	48.17 ± 2.1	47.1 ± 2.1	22.1 ± 1.8	21.6 ± 2.4	23.7 ± 1.6	22.4 ± 1.9	22.4 ± 2.1	21.5 ± 1.8

*Values are means ± SE. VO_2_, rate of oxygen uptake; HR, heart rate; V_E_/VCO_2_, ventilatory equivalent for carbon dioxide; P_ET_CO_2_, end-tidal partial pressure of carbon dioxide; SpO_2_, oxyhemoglobin saturation; V_E_, minute ventilation; V_T_, tidal volume; SVC, slow vital capacity measured prior to start of exercise; f_R_, respiratory frequency; IC, inspiratory capacity; IRV, inspiratory reserve volume*.

The relative contributions of breathlessness [0.9% saline, 41.9 ± 3.4%; 40 mg furosemide, 40.2 ± 3.6%; 120 mg furosemide, 39.4 ± 4.0% (*p* = 0.876)], leg discomfort [0.9% saline, 55.4 ± 4.1%; 40 mg furosemide, 54.4 ± 4.2%; 120 mg furosemide, 51.7 ± 4.6% (*p* = 0.746)] and chest tightness [0.9% saline, 2.4 ± 1.5%; 40 mg furosemide, 0.8 ± 0.8%; 120 mg furosemide, 6.0 ± 3.3% (*p* = 0.170)] to exercise cessation were not significantly different across treatments. The distribution of reasons for stopping exercise was similar across treatments: breathlessness [0.9% saline, *n* = 2; 40 mg furosemide, *n* = 4; 120 mg furosemide, *n* = 3 (*p* = 0.683)]; leg discomfort [0.9% saline, *n* = 8; 40 mg furosemide, *n* = 9; 120 mg furosemide, *n* = 8 (*p* = 0.941)]; combination of breathlessness and leg discomfort [0.9% saline, *n* = 14; 40 mg furosemide, *n* = 10; 120 mg furosemide, *n* = 10 (*p* = 0.410)]; and other (0.9% saline, *n* = 0; 40 mg furosemide, *n* = 1; 120 mg furosemide, *n* = 3). The selection frequencies of the qualitative descriptors of breathlessness at end-exercise were also similar across treatments: “*My breath does not go in all the way”* [0.9% saline, 65.2%; 40 mg furosemide, 69.6%; 120 mg furosemide, 65.2% (*p* = 0.937)]; “*Breathing in requires effort”* [0.9% saline, 91.3%; 40 mg furosemide, 87.0%; 120 mg furosemide, 73.9% (*p* = 0.245)]; “*I feel a need for more air”* [0.9% saline, 91.3%; 40 mg furosemide, 82.6%; 120 mg furosemide, 87.0% (*p* = 0.682)]; “*My breathing is heavy”* [0.9% saline, 95.7%; 40 mg furosemide, 100%; 120 mg furosemide, 95.7% (*p* = 1.00)]; “*I cannot take a deep breath in”* [0.9% saline, 60.9%; 40 mg furosemide, 60.9%; 120 mg furosemide, 78.3% (*p* = 0.352)]; “*My chest feels tight”* [0.9% saline, 95.7%; 40 mg furosemide, 91.3%; 120 mg furosemide, 91.3% (*p* = 0.806)]; “*My breathing requires more work”* [0.9% saline, 91.3%; 40 mg furosemide, 87.0%; 120 mg furosemide, 91.3% (*p* = 0.853)]; “*I feel a hunger for more air”* [0.9% saline, 87.0%; 40 mg furosemide, 87.0%; 120 mg furosemide, 82.6% (*p* = 0.890)]; “*I feel that my breathing is rapid”* [0.9% saline, 91.3%; 40 mg furosemide, 78.3%; 120 mg furosemide, 91.3% (*p* = 0.317)]; “*My breathing feels shallow”* [0.9% saline, 78.3%; 40 mg furosemide, 73.9%; 120 mg furosemide, 69.6% (*p* = 0.798)]; and “*I cannot get enough air in”* [0.9% saline, 82.6%; 40 mg furosemide, 69.6%; 120 mg furosemide, 73.9% (*p* = 0.579)].

### Debriefing

There was no statistically significant difference in the percentage of participants that correctly identified the visit at which they inhaled the 0.9% saline, 40 mg furosemide, and 120 mg furosemide solutions: 45.8, 41.7, and 62.5%, respectively (*p* = 0.311 by chi-squared test).

## Discussion

The primary finding of this study is that inhalation of nebulized furosemide at doses of 40 and 120 mg had no demonstrable effect on ratings of perceived breathlessness during constant-load cycle endurance exercise testing in the setting of abnormal restrictive constraints on V_T_ expansion by external thoracic restriction in healthy men.

The results of our study are in contrast to those of earlier studies by: (1) Nishino et al. (2000) who reported that inhalation of nebulized furosemide (40 mg) compared with nebulized 0.9% saline decreased the intensity of breathlessness induced by voluntary breath-holding and by a combination of hypercapnia and inspiratory resistive loading in 12 healthy subjects; (2) Minowa et al. (2002) who demonstrated that the magnitude of increase in breathlessness intensity ratings during hypercapnic hyperpnea was not significantly different after vs. before inhalation of nebulized 0.9% saline, but was significantly reduced after vs. before inhalation of nebulized furosemide (40 mg) in 10 healthy subjects; (3) Moosavi et al. (2007) who demonstrated that the magnitude of the pre- to post-dose decrease in breathlessness intensity ratings was marginally greater for nebulized furosemide (40 mg) compared with nebulized 0.9% saline (*p* = 0.052) in 10 healthy subjects; and (4) Ong et al. (2004) and Jensen et al. (2008) who found that breathlessness intensity ratings were significantly lower (by ~20%) at a standardized submaximal time during constant-load cycle endurance exercise testing after inhalation of nebulized furosemide (40 mg) compared with nebulized 0.9% saline in 19 and 20 adults with COPD, respectively. Potential reasons for the discrepant results between these earlier studies and our own [with the obvious exception of our comparatively larger sample size (*n* = 24)] are discussed in the *Methodological Considerations* section below.

Clear evidence of diuresis was apparent after our participants inhaled the 120 mg nebulized furosemide solution compared with both 40 mg furosemide and 0.9% saline solutions. Nevertheless, inhalation of the 120 mg nebulized furosemide solution had no effect on exertional breathlessness, suggesting that systemic absorption of furosemide from the gastrointestinal tract cannot explain earlier reports of breathlessness relief following inhalation of nebulized furosemide (40 mg) compared with 0.9% saline (Nishino et al., 2000; Minowa et al., 2002; Ong et al., 2004; Moosavi et al., 2007; Jensen et al., 2008).

Our findings confirm and extend those of earlier studies by: (1) Wilcock et al. (2008) who found that intensity ratings of perceived breathlessness during arm exercise were not significantly different following inhalation of nebulized furosemide (40 mg) compared with nebulized 0.9% saline in 15 symptomatic patients with lung cancer or mesothelioma; (2) Panahi et al. (2008) who demonstrated that the magnitude of the pre- to post-dose decrease in breathlessness intensity ratings recorded at rest was not significantly different for inhalation of nebulized furosemide (40 mg) compared with nebulized 0.9% saline in 41 adults with irreversible obstructive airway disease due to sulfur mustard gas exposure; and (3) Laveneziana et al. (2008) who reported that inhalation of nebulized furosemide (40 and 80 mg) compared with nebulized 0.9% saline had no effect on intensity ratings of breathlessness during expiratory flow-limited incremental cycle CPET in nine healthy adults.

### Methodological considerations

As reviewed in the introduction, relief of breathlessness following inhalation of nebulized furosemide has been mechanistically linked to altered pulmonary vagal afferent activity from PSRs (most likely SARs), presumably mimicking greater V_T_ expansion (Nishino et al., 2000; Sudo et al., 2000; Nehashi et al., 2001; Moosavi et al., 2007; Nishino, 2009). On this basis, we reasoned that the potential dose-response effect of nebulized furosemide on breathlessness might be uniquely revealed during constant-load CPET in the presence of an external thoracic restriction when V_T_ expansion (and presumably also activation of SARs) is reduced and breathlessness is (i) severely intense and unpleasant, (ii) described as a heightened sense of “unsatisfied inspiration” and (iii) identified as a main exercise-limiting symptom (Harty et al., 1999; O'Donnell et al., 2000; Mendonca et al., 2014; Kotrach et al., 2015). By studying healthy men as opposed to symptomatic adults with advanced disease, we also minimized the potentially confounding influences of psycho-physiological comorbidities, skeletal muscle deconditioning/dysfunction, hypoxemia, hypercapnia, concomitant medication use, etc. on exertional breathlessness, presumably increasing our ability to demonstrate a potential dose-response effect of nebulized furosemide on exertional breathlessness. Nevertheless, neither 40 nor 120 mg doses of nebulized furosemide had an effect on exertional breathlessness compared with nebulized 0.9% saline.

It could be argued that external thoracic restriction may have masked a potential effect of nebulized furosemide on exertional breathlessness *via* stimulation of pulmonary irritant receptors and/or RARs by way of alveolar collapse (atelectasis) and/or breathing at abnormally low lung volumes, respectively. However, studies by Sant'Ambrogio et al. (1993) and Sudo et al. (2000) showed that inhalation of nebulized furosemide inhibited the activity of laryngeal irritant receptors to stimulation by inhalation of low-chloride solutions in anesthetized, spontaneously breathing dogs and suppressed the activity of RARs during lung inflation in anesthetized rats, respectively. On the basis of these observations, we contend that our use of external thoracic restriction likely served to increase the probability of demonstrating an effect of nebulized furosemide compared with nebulized 0.9% saline on exertional breathlessness. Furthermore, if CWS caused a meaningful degree of atelectasis (and attendant ventilation-perfusion mismatching), then it is reasonable to assume that the ventilatory equivalent for carbon dioxide (V_E_/VCO_2_)–an index of exercise ventilatory efficiency–would be elevated during exercise with vs. without CWS; however, this does not appear to be the case since earlier studies by O'Donnell et al. (2000), Mendonca et al. (2014), and Kotrach et al. (2015) reported no statistically significant effect of CWS sufficient to reduce vital capacity by ~20–35% of its baseline (unrestricted) value on the V_E_/VCO_2_ response to symptom-limited incremental and constant-load cycle CPET.

By design, CWS decreased SVC recorded prior to CPET at each treatment visit by an average of 21–22% of its baseline (unrestricted) value and to within an average of ≤1% of the target SVC value recorded prior to CPET at *Visit 1*. The intra-subject, between-day (or between-treatment) coefficient of variability in the SVC value recorded prior to CPET was very low at just 2.2 ± 0.2%, confirming our ability to reproducibly restrict lung volumes *via* CWS across treatment visits. Thus, it is unlikely that intra-subject, between-day (or between-treatment) variability in the extent of external thoracic restriction confounded our ability to demonstrate a potential dose-response effect of nebulized furosemide on exertional breathlessness.

Even though our participants were asked to rate the intensity and unpleasantness of perceived breathlessness separately from the intensity of perceived chest tightness, we cannot rule out the possibility that conflation of these ratings concealed a potential dose-response effect of nebulized furosemide on exertional breathlessness. However, the percentage contributions of chest tightness and of breathlessness to exercise cessation were markedly different across treatments (≤6% vs. 39–42%, respectively), even though Borg CR10 scale ratings of these two symptoms were quantitatively similar during CPET. Thus, it seems unlikely that the null results of our study can be readily explained by conflation of ratings of chest tightness and of breathlessness.

Several steps were taken to optimize delivery of nebulized furosemide to the airways and lungs. First, we studied healthy men with normal spirometry (and presumably also normal airway geometry) and without airway inflammation, obstruction and/or secretions (Newman, 1985). Second, nebulized solutions were delivered through the mouth alone (albeit with an open facemask) during deep and slow tidal inspirations (Newman, 1985; Everard et al., 1993). Third, we used a compressed air (jet) nebulizer that produced particles with a mass median diameter of ~5 μm, which are within the “respirable range” for therapeutic aerosols (Newman, 1985). Fourth, we used a nebulized dose of furosemide (120 mg) that was 3-fold higher than the 40 mg dose used in most previous studies (Nishino et al., 2000; Minowa et al., 2002; Ong et al., 2004; Moosavi et al., 2007; Jensen et al., 2008; Laveneziana et al., 2008; Panahi et al., 2008; Wilcock et al., 2008; Sheikh Motahar Vahedi et al., 2013). Despite these considerations and for reasons described in detail elsewhere (Newman, 1985), it is likely that only a small fraction (~10%) of the available furosemide was actually deposited into the airways and lungs of our participants during nebulization. Thus, the possibility exists that the null results of our study reflect, at least in part, limited delivery of nebulized furosemide into the airways and lungs of our participants; that is, nebulized furosemide failed to reach and subsequently act on PSRs. However, earlier studies reporting a beneficial effect of just 40 mg of nebulized furosemide compared with nebulized 0.9% saline on the perception of breathlessness in health (Nishino et al., 2000; Minowa et al., 2002; Moosavi et al., 2007) and COPD (Ong et al., 2004; Jensen et al., 2008; Sheikh Motahar Vahedi et al., 2013) all employed compressed air (jet) nebulizers with similar performance characteristics (i.e., particle size range, nebulization rate) as the one used in our study. Furthermore, the results of studies by Morelot-Panzini et al. (2018) and Banzett et al. (2018) indicated that optimal delivery of nebulized furosemide at doses of 40 and 80 mg using a mechanical ventilator at strictly controlled inspiratory flow rates (300–500 ml/s) and levels of V_T_ expansion (15% of predicted vital capacity) did not produce more consistent and/or greater relief of laboratory-induced breathlessness compared with nebulized 0.9% saline in healthy adults. Thus, it seems unlikely that factors related to suboptimal delivery of nebulized furosemide to the airways and lungs can explain the null results of our study.

According to the results of their randomized, single-blind, parallel group study of patients admitted to the hospital for an exacerbation of COPD, Khan and O'Driscoll (2004) concluded that nebulized 0.9% saline cannot be used as an inert placebo in studies assessing relief of breathlessness with therapeutic aerosols. In support of this conclusion, studies by Panahi et al. (2008), Banzett et al. (2018), and Morelot-Panzini et al. (2018) have reported statistically significant pre- to post-dose relief of breathlessness for both nebulized 0.9% saline and nebulized furosemide (40–80 mg). In each of these studies, the magnitude of relief produced by nebulized 0.9% saline was comparable to that produced by nebulized furosemide (Panahi et al., 2008; Banzett et al., 2018; Morelot-Panzini et al., 2018). O'Donnell et al. (2017) have provided evidence that relief of breathlessness following inhalation of nebulized 0.9% saline can be largely explained by participants' expectation of a treatment effect (i.e., “placebo effect”). As part of the informed consent procedure, the men who participated in our study were told that our primary objective was to determine whether inhalation of nebulized furosemide decreases the perception of breathlessness during exercise. In light of the above, and even though there was no statistically significant difference in the percentage of participants that identified correctly the nebulized solution they received at study *Visits 2, 3, and 4*, we cannot rule out the possibility that the null results of our study may be due, at least in part, to a “placebo effect.”

While the duration of action of nebulized furosemide is not known, Moosavi et al. (2007) reported that relief of breathlessness following single-dose inhalation of nebulized furosemide (40 mg) compared with nebulized 0.9% saline dissipated after an average of 1-hr. In our study, constant-load CPETs across all three treatments were completed within 85–95 min and 50–55 min from the start and from the end of the nebulization period, respectively. It follows that the null results of our study may be due to diminution of furosemide's effect on pulmonary vagal afferent activity from PSRs. However, a randomized, placebo-controlled, crossover study by Novembre et al. (1995) found that doubling the dose of nebulized furosemide from 15 to 30 mg prolonged (but did not enhance) its preventive effect on exercise-induced asthma in children from at least 2-h to at least 4-h. Thus, it seems unlikely that the lack of effect of 40 mg and especially 120 mg of nebulized furosemide on exertional breathlessness in our study can be explained by diminution of furosemide's effect on pulmonary vagal afferent activity from PSRs.

In conclusion, inhalation of nebulized furosemide at doses of 40 and 120 mg did not alleviate the perception of breathlessness during exercise in healthy men, at least not under the experimental conditions of our study.

## Author contributions

MW-F, JB, BS, and DJ: conceived the study; MW-F, AW, AM, NM, AA, and DJ: contributed to the collection and/or analysis of data; MW-F and DJ: wrote the manuscript with critical input from all other authors; All authors read and approved the final version of the manuscript.

### Conflict of interest statement

The authors declare that the research was conducted in the absence of any commercial or financial relationships that could be construed as a potential conflict of interest.
